# Supplementary material for: The Role of Knowledge, Attitude, Confidence, and Sociodemographic Factors in COVID-19 Vaccination Adherence among Adolescents in Indonesia: A Nationwide Survey
Source: Vaccines (Basel). 2022 Sep 7;10(9):1489. doi: 10.3390/vaccines10091489 (PMC9505584; doi:10.3390/vaccines10091489)
Supplement: Supplementary file 1 [file vaccines-10-01489-s001.zip › vaccines-1837829-supplementary.pdf]

Supplementary Materials

Instruments

Knowledge of COVID-19 Vaccine

1. COVID-19 vaccines use inactivated coronavirus as the antigen
2. COVID-19 vaccines use genetic material from coronavirus as the active ingredient
3. COVID-19 vaccine stimulates our body to produce antibody, T cells and memory cells to combat COVID-19 infection
4. COVID-19 vaccine protects the receiver from getting COVID-19 infection
5. COVID-19 vaccination may protect other people who do not receive vaccine
6. Vaccine production involves animal study, 3 phases of clinical trials that cover thousands of people and evaluated by the authority to ensure the vaccine efficacy and safety
7. COVID-19 vaccines will be given via injection
8. COVID-19 vaccines do not have adverse effects
9. Everyone including children can receive COVID-19 vaccination
10. COVID-19 vaccine can also protect us from influenza

Attitude toward vaccines

1. "Do you worry about being infected with COVID-19?" (No/Fair/Very much)
2. "Have you heard of COVID-19 vaccines previously such as any information about COVID-19 vaccines, including both positive and negative news, vaccine development, safety and efficacy of vaccine via various channels (e.g., radio, television, telephone)?" (No/Yes)
3. "Do you think COVID-19 vaccines could protect you from COVID-19?" (No/Yes/No idea);
4. "How safe do you think COVID-19 vaccines are?" (Not safe with obvious side effects/No idea/Safe with no or minimal side effects);
5. "Would you encourage your family and friends to get vaccinated with the COVID-19 vaccine?" (No/Yes/No idea).

6. An extra question was asked to examine participants' behavior toward COVID-19 vaccines: "Do you intend to be vaccinated against COVID-19 in the future?" (No/Yes).

Willingness

"If there was the possibility to get vaccinated against the coronavirus, would you do it?" rated on a 5-point scale from 1="no way" to 5="definitely".

Confidence in Covid-19 vaccines

1. do you think you will be infected with COVID-19 over the next 12 months?
- Definitely
- Probably
- Possibly
- Probably not
- Definitely not
- Don't know

2. The COVID-19 vaccine is likely to:
- Work for almost everyone
- Work for most people
- I am unsure how many people it will work for
- Not work for most people
- Not work for anyone
- Don't know

3. The COVID-19 vaccine is likely to:
- Definitely work for me
- Probably work for me
- May or may not work for me
- Probably not work for me
- Definitely not work for me

- Don't know

4. If I get the COVID-19 vaccine it will be:

- Really helpful for the community around me
- Helpful for the community around me
- Neither helpful nor unhelpful for the community around me
- Unhelpful for the community around me
- Really unhelpful for the community around me
- Don't know

5. If individuals like me get the COVID-19 vaccine it will:

- Save a large number of lives
- Save some lives
- Have no impact
- Lead to more deaths
- Lead to a large number of deaths
- Don’t know

6. The spreed of developing and testing the vaccine means it will be

- Really good
- Good
- Will not affect how good or bad it is
- Bad
- Really bad
- Don't know

7. The spreed of developing and testing the vaccine means it will be

- Really safe
- Safe
- It will not affect how safe it is
- Unsafe

- Really unsafe

8. If many people do not get the vaccine this:

- Will be dangerous
- May be dangerous
- Will have no consequences at all
- May be good
- Will be good
- Don't know

9. l expect that receiving the vaccine will be:

- Hardly noticeable
- A little unpleasant
- Moderately unpleasant
- Painful
- Extremely painful
- Don't know

10. The side effects for people of getting the COVID-19 vaccine will be:

- None
- Mild
- Moderate
- Life-threatening
- Don't know

11. The COVID-19 vaccine will:

- Greatly strengthen my immune system
- Strengthen my immune system
- it will neither strengthen nor weaken my immune system
- Weaken my immune system
- Greatly weaken my immune system

- Don't know

12. Taking the COVID-19 vaccine:

- Will give me complete freedom to get on with life just as before
- Will give me greater freedom
- Will have no effect on my freedom
- Will restrict my freedom
- Will completely restrict my freedom to get on with Me
- Don't know

13. Getting the vaccine is a sign of:

- Great personal strength
- Personal strength
- Not a sign of personal strength or weakness
- Personal weakness
- Great personal weakness
- Don't know

14. Taking a new COVID-19 vaccine will make me feel like a guinea pig:

- Do not agree
- Agree a little
- Agree moderately
- Agree a lot
- Completely agree
- Don't know
